# MUSCULOSKELETAL ALTERATIONS OF ORTHOPEDIC INTEREST IN MUCOPOLYSACCHARIDOSES

**DOI:** 10.1590/1413-785220253303e290442

**Published:** 2025-12-01

**Authors:** Marcos Almeida Matos, Paloma Silva Lopes

**Affiliations:** 1 Escola Bahiana de Medicina e Saude Pública (EBMSP), Bahia, BA, Brazil.

**Keywords:** Mucopolysaccharidoses, Limb Deformities, Congenital, Functional Independence, Mucopolissacaridoses, Deformidades Congênitas dos Membros, Independência Funcional

## Abstract

Mucopolysaccharidoses (MPS) are lysosomal storage diseases characterized by the improper accumulation of glycosaminoglycans due to genetic deficiencies in specific enzymes. This accumulation leads to progressive cellular and tissue dysfunction, affecting multiple systems, particularly the musculoskeletal system, resulting in multiple dysostoses with deformities in the spine, thorax, and limbs. Clinically, MPS manifests as recurrent respiratory infections, sleep apnea, severe cardiac lesions, and hydrocephalus, among other symptoms. Bone and joint alterations markedly impair motor function and the quality of life of patients. However, early diagnosis can be achieved by identifying osteoarticular signs, which are crucial for the immediate initiation of treatment. Despite the well-pronounced skeletal manifestations, there is a lack of recognition among specialists, indicating the need for detailed reviews for medical professionals, especially orthopedists, radiologists, pediatricians, and geneticists. This study aims to highlight the orthopedic alterations of MPS, emphasizing their radiographic aspects, which are essential for suspicion, differential diagnosis, and correct identification, contributing to better management of these diseases. *Level of Evidence V; Review Article.*

## RESUMO

Mucopolissacaridoses (MPS) são doenças de armazenamento lisossômico caracterizadas pelo acúmulo inadequado de glicosaminoglicanos, devido à deficiência genética de enzimas específicas. Esse acúmulo provoca disfunção celular e tecidual progressiva, afetando múltiplos sistemas, especialmente o sistema osteoarticular, resultando em disostose múltipla com deformidades na coluna, tórax e membros. Clinicamente, as MPS manifestam-se por infecções respiratórias recorrentes, apneia do sono, lesões cardíacas graves, hidrocefalia, entre outros sintomas. As alterações ósseas e articulares comprometem significativamente a funcionalidade motora e a qualidade de vida dos pacientes. No entanto, o diagnóstico precoce pode ser feito pela identificação de sinais osteoarticulares, condição fundamental para o início do tratamento imediato. Apesar das manifestações esqueléticas serem bem pronunciadas, há uma carência de reconhecimento entre especialistas, indicando a necessidade de revisões detalhadas para profissionais da área médica, especialmente ortopedistas, radiologistas, pediatras e geneticistas. Este estudo visa evidenciar as alterações ortopédicas das MPS, destacando seus aspectos radiográficos, essenciais para a suspeita, diagnóstico diferencial e identificação correta, contribuindo para um melhor manejo dessas doenças. *Nível de Evidência V; Artigo de Revisão.*

## INTRODUCTION

Mucopolysaccharidoses are lysosomal storage diseases characterized by the non-degradation and consequent improper storage of glycosaminoglycans in the lysosomes.^1^ Degradation does not occur due to genetic defects that result in the lack of enzymes responsible for the catabolism of glycosaminoglycans.^2^ Each mucopolysaccharidosis is caused by a mutation that determines the missing enzyme and the mucopolysaccharide that will accumulate (Table 1).^1,2^ This lysosomal accumulation leads to gradual and progressive cellular and tissue dysfunction, characterized by multisystemic involvement and generally lethal for individuals affected by mucopolysaccharidoses.^3^

**Table 1:** Classification of MPS, according to enzyme deficiency and accumulated substrate (GAG).

Type of MPS	Eponym	Enzyme deficiency	Substrate
MPS I	Scheie, Hurler–Scheie, Hurler syndromes	α-L-iduronidase	DS/HS
MPS II	Hunter syndrome	Iduronate-2-sulfatase	DS/HS
MPS III-A	Sanfilippo A syndrome	Heparan-N-sulfatase	HS
MPS III-B	Sanfilippo B syndrome	α-N-acetylglucosaminidase	HS
MPS III-C	Sanfilippo C syndrome	Acetyl-CoA-α-glucosaminide acetyltransferase	HS
MPS III-D	Sanfilippo D syndrome	N-acetylglucosamine 6-sulfatase	HS
MPS IV-A	Morquio A syndrome	Galactose-6-sulfatase	KS
MPS IV-B	Morquio B syndrome	β-galactosidase	KS
MPS VI	Maroteaux–Lamy syndrome	N-acetylgalactosamine 4-sulfatase	DS
MPS VII	Sly disease	β-glucuronidase	DS/HS
MPS IX	Natowicz disease	Hyaluronidase	HA

Source: Adapted from Muenzer J.^1^ Caption: HA – Hyaluronic acid; coA – coenzyme A; DS – Dermatan sulfate; HS – Heparan sulfate; MPS – Mucopolysaccharidosis; KS – Keratan sulfate; α – alpha; β – beta.

Mucopolysaccharidoses (MPS) are rare and serious diseases that predominantly affect the lungs, cardiorespiratory system, central and peripheral nervous system, and in a very specifically way, the osteoarticular system.^1^ The involvement of the musculoskeletal system in mucopolysaccharidoses is known as multiple dysostosis and results in alterations of the spine, chest, and limbs. Clinically, MPS is characterized by recurrent respiratory infections, snoring, sleep apnea, severe valvular heart lesions, hydrocephalus, spasticity, among other injuries.^4,5^

The involvement of the bones and joints significantly contributes to the loss of motor functionality and the quality of life of patients, leading to even greater human suffering.^6^ However, early diagnosis can be achieved by recognizing the osteoarticular signs of the disease, which are, generally, constant and precocious, and may even exist before the first year of life.^7^ Recognizing skeletal signs of MPS is of particular importance, as many MPS now have a baseline treatment whose effectiveness depends directly on early medication initiation.^7,8^

Despite the musculoskeletal signs being multiple and well pronounced, there is evidence that even specialists in skeletal dysplasias are not able to diagnose MPS by observing such signs.^9,10^ This points to the need for reviews that are capable of detailing and popularizing these bone alterations among general practitioners, but, especially, orthopedists, radiologists, pediatricians, and geneticists. For this reason, the present study aims to thoroughly review the orthopedic alterations of mucopolysaccharidoses, emphasizing their radiographic aspects.

## PATHOLOGY

Glycosaminoglycans (GAGs) accumulate in both intracellular and extracellular compartments.^11^ These compounds bind to proteins to form proteoglycans, which have a structural function. GAGs also have functions in various cellular processes, such as adhesion, transduction, and activation of specific inflammatory pathways.^11,12^ The accumulation of GAGs is involved in the apoptosis of chondrocytes and in the increase of TNF-_α_ levels, which may be the main factors responsible for the musculoskeletal manifestations of the disease.^11,12^

The involvement of bones, cartilage, synovial fluid, capsule, ligaments, tendons, and other periarticular tissues is a common finding in all types of mucopolysaccharidoses.^12-14^ Endochondral and pseudomembranous bone growth as well as bone remodeling processes are affected, leading to diaphyseal, metaphyseal, and epiphysial changes.^13,14^

The accumulation of GAGs in the periarticular tissues associated with epiphyseal changes leads to stiffness, contractures, and increased joint volume, causing the so-called "dry arthritis," where there is no associated inflammatory process.^15^ The deposition of GAGs in the flexor tendons, with thickening of the retinaculum and pulley, associated with flexion stiffness of the interphalangeal joints, is responsible for the characteristic claw hand of MPS.^16,17^ The thickening of the flexor retinaculum and tendon sheaths in the carpal tunnel leads to compression of the median nerve at the wrist (carpal tunnel syndrome).^18^

## Radiographic Evaluation

Clinical and laboratory diagnosis is the gold standard in cases of MPS, including enzymatic dosage and genetic sequencing using skeletal dysplasia panels.^10^ Conventional radiography is essential for suspicion, differential diagnosis, and most of the time, it is possible to identify radiographic characteristics that lead to the correct diagnosis.^10,19^ A complete skeletal radiographic evaluation should be performed as isolated images can lead to incorrect diagnoses.^10,19^ The best approach is to perform the standard screening for MPS, which includes AP and lateral skull radiographs, AP and lateral thoracolumbar spine, AP chest, AP pelvis, AP upper limb panoramic, AP lower limb panoramic, and AP left hand.^19,20^ In this study, the main radiographic aspects of the orthopedic alterations of mucopolysaccharidoses will be presented.

## Long bone and limbs deformities

### Upper limb

**Figure 1:**
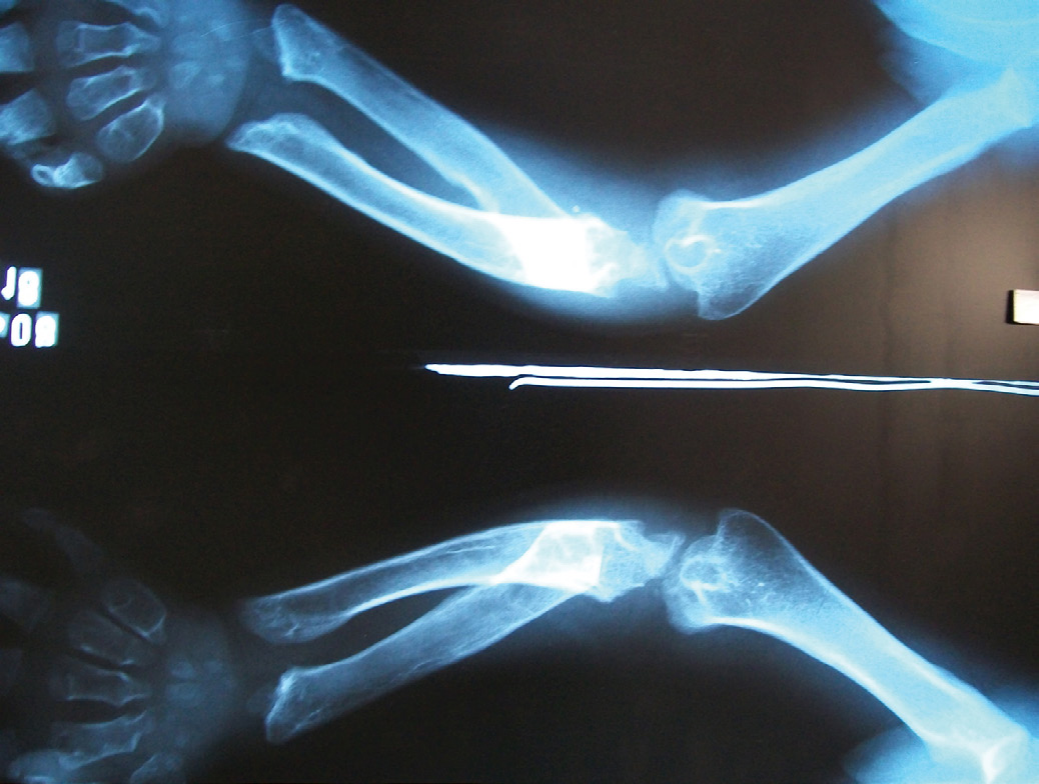
Shortening of the radius and ulna with irregularities and abnormal curvature, associated with cortical thinning and medullary rarefaction (osteopenia), creating an inflated appearance; distal and proximal dysplasia of both bones is also noted, with widening and irregularities of the metaphyses, as well as convergent inclination of the distal metaphyses.

Source: Author's.

Dysfunction in the endochondral growth of long bones causes decreased length with a relative increase in width, resulting in overall shortening of the upper and lower limbs with subsequent short stature.^13,14^ There is metaphyseal widening that creates the appearance of periarticular edema which is called "dry arthritis"; the physis is irregular and hypoplastic with erratic and misdirected growth, originating diaphyseal tortuosity that results in deformities such as the so-called "Madelung-like" in the wrist (Figure 1). The alteration in remodeling and pseudomembranous growth associated with changes in endochondral growth causes thinning of the cortices, osteopenia, bone fragility, and contributes to the irregularities observed in the longitudinal axis of the diaphysis.^13,14^

**Figure 2:**
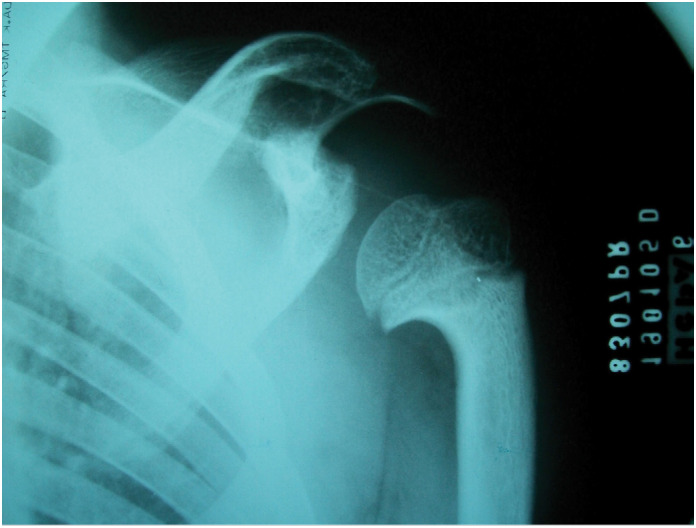
Shortened, widened, and irregular clavicle with increased curvature; dysplastic glenoid cavity; proximal humeral metaphyseal widening with abnormal notching in the medial region and varus deformity of the neck (shepherd's crook).

Source: Author's.

The shoulder joint presents hypoplasia of the proximal humeral epiphysis with a wide and shallow glenoid cavity. There is cortical thinning with medullary widening, and the proximal humerus is in varus with prominence of the greater tubercle exhibiting a medial notch that gives it the appearance of a "shepherd's crook" deformity.^21^ The clavicle is thick and hypoplasic in its lateral portion, and the scapula is also relatively small and malformed.^20,21^ (Figure 2).

**Figure 3:**
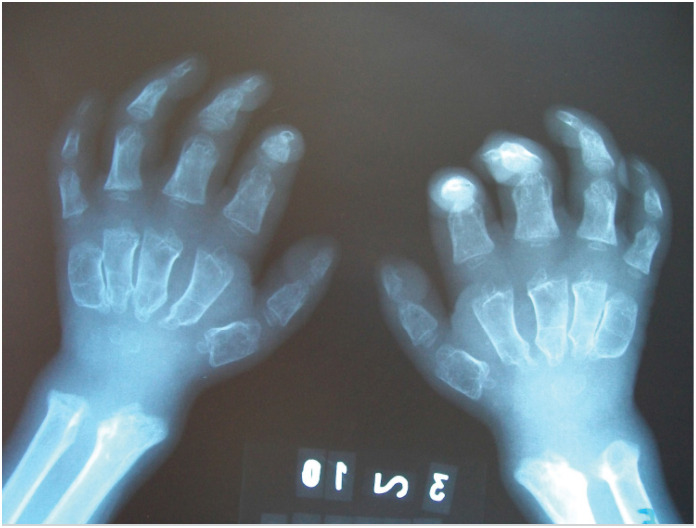
Radial and ulnar dysplasia with metaphyseal and epiphyseal irregularities, with ends inclined toward each other (Madelung-like deformity); carpal bones with delayed and irregular ossification; metacarpal bones shortened, widened, irregular, with thinned cortices and conical-shaped proximal ends (beak-shaped); malformed phalanges with widened bases and narrow, rounded apices (bullet-shaped).

Source: Author's.

The wrist has hypoplasia of the distal radius and ulna with widening of both metaphyses, which are inclined towards each other (Madelung-like deformity), giving the appearance of a "V"-shaped carpus. The carpal bones are irregular, small, with late ossification of the nuclei. The metacarpals are arranged in a fan shape and present proximal thinning sometimes with a small beak and distal widening.^21^ Like other long bones, the metacarpals are also shorter and wider, the physes are tortuous, and the epiphyses are hypoplastic, irregular and and of late appearance. The phalanges have the characteristic appearance of a bullet, being wider proximally and tapered with a smooth rounding distally.^21^ (Figure 3).

### Lower limb

**Figure 4:**
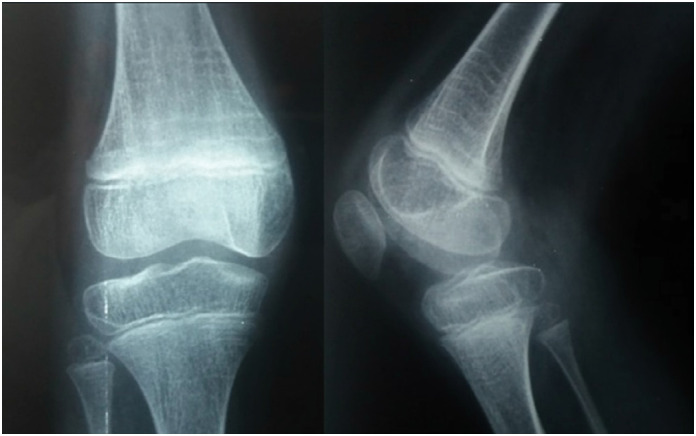
Radiological image of the hip showing flattened and malformed femoral head associated with femoral neck valgus. There are also acetabular dysplasia and head subluxation.

Source: Author's.

Hip dysplasia in MPS is usually very severe and can lead to subluxation, dislocation, late degenerative joint disease, or even femoral head osteonecrosis.^19,20,22,23^ Most patients present with increased cartilage associated with a shallow and poorly developed acetabulum in its lateral portion, which is also poorly ossified, producing extrusion of the femoral head with increased acetabular and increased Riemers indices.^19,20,22,23^ The iliac wing has a fan-shaped and rounded appearance with lateral inclination and is tapered distally. The ossification of the femoral head is late, irregular, with the medial portion poorly developed and gradually fragmented, resembling Perthes disease.^10^ The proximal femur is in valgus with an enlarged neck, producing a Shenton line rupture. (Figure 4).

**Figure 5:**
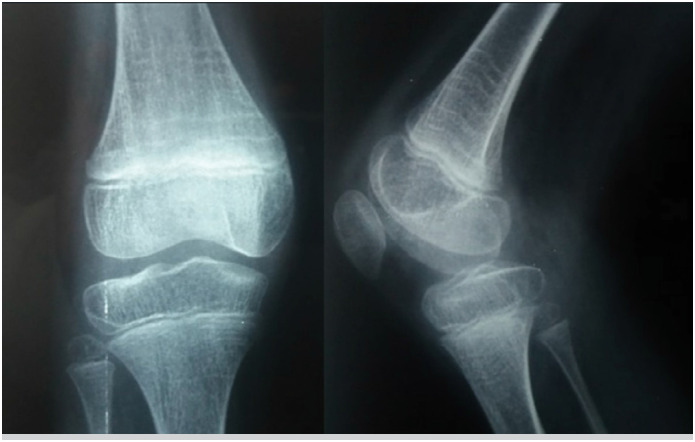
Radiological image of the knee in PA and lateral views, showing hypoplasia of the lateral part of the tibial epiphysis – valgus knee.

Source: Author's.

**Figure 6:**
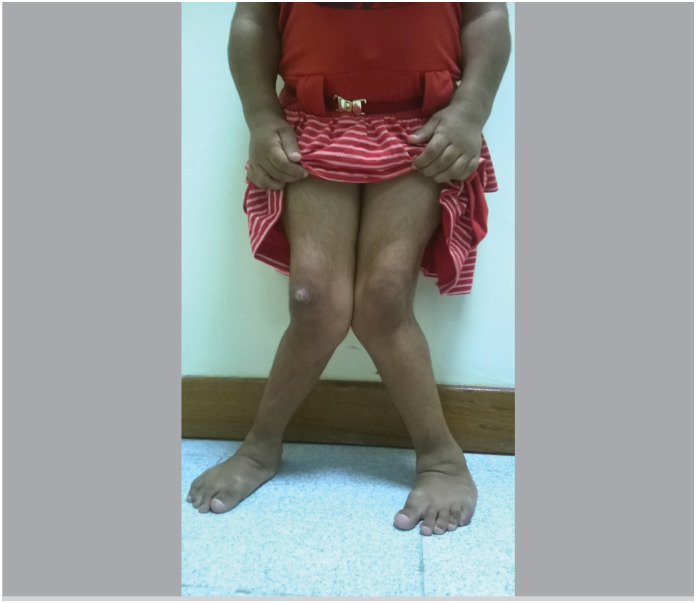
Clinical characteristic of valgus knee (knock knee) and plano-valgus foot deformity with flexed toes – claw deformity.

Source: Author's.

**Figure 7:**
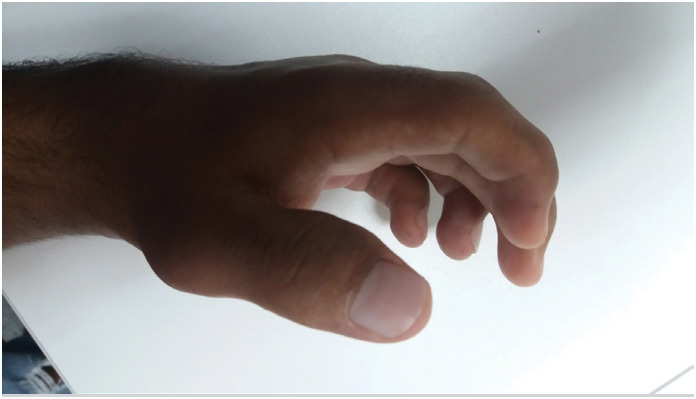
Clinical appearance of the hand in a claw-like posture with flexion of the distal and proximal interphalangeal joints, and the metacarpophalangeal joints.

Source: Author's.

The tibia has hypoplasia of the lateral part of the epiphysis, producing the characteristic valgus knee, especially in MPS type IV, which usually develops after two years of age and progresses slowly, causing joint degeneration.^19,23^ The feet in MPS can vary between planovalgus patterns with twisted toes^24^ or cavovarus with equinus; there is lateral hypoplasia of the distal tibial physis, and the fibula is curved towards the tibia, often with absence or hypoplasia of the distal ossification center.^24^ Gradually, the feet become clinically equinus and acquire forefoot widening that makes it difficult to fit into shoes, while shortening and overlapping of the foot rays are also common.^24,25^ As previously described, the accumulation of GAGs in tendons and tendon sheaths gradually produces flexion deformity of the toes, leading to similar claw deformity seen in the hand.^26^ (Figures 5, 6 and 7).

## Spine deformities

### Cervical spine

The craniocervical junction stenosis is a common finding in MPS. This alteration occurs due to multiple factors that ultimately are responsible for stenosis, which can lead to spinal cord compression, myelopathy, and signs and symptoms of upper motor neuron compromise.^19,27,28^ The accumulation of GAGs in the ligaments and meninges produces tissue thickening, particularly in the periodontal region (thickened dura and hypertrophied ligamentum flavum). Other findings that contribute to stenosis are platybasia, hypoplasia of both odontoid and C^1^ arch, disc protrusion, and basilar invagination.^19,27,28^

**Figure 8:**
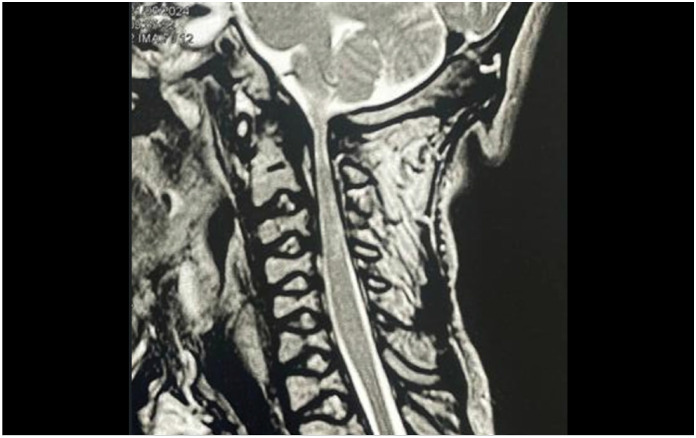
MRI image of the cervical spine showing spinal cord compression and myelopathy.

Source: Author's.

Atlantoaxial instability, which is particularly found in MPS type IV, is another significant factor associated with craniocervical injury.^19,27^ In the case of other forms of MPS, tissue thickening causes cervical stiffness, which relatively protects against craniocervical instability. However, patients with MPS IV present C^1^-C^2^ hypermobility, making them especially susceptible to spinal cord injury. Baseline treatment with enzyme replacement therapy reduces the accumulation of GAGs, which is also a risk factor that should be monitored by cervical spine magnetic resonance imaging.^27,29^ (Figure 8)

### Thoracolumbar spine

**Figure 9:**
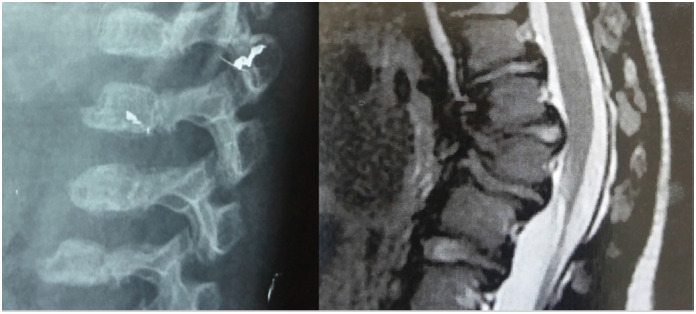
In this figure, it is possible to see the typical shape of the vertebrae (betta fish) on the X-ray, as well as the retropulsion of the intervertebral discs causing stenosis of the spinal canal at the kyphosis apical vertebra.

Source: Author's.

The vertebral spine is characterized by platyspondyly, anterior beaking of the vertebral body, with maintained or increased disc height. The beak is located anteroinferiorly in MPS I and centrally in MPS IV.^19,30^ The vertebrae also present a posterior notch in the body,^31^ and their overall shape resembles the profile image of a "betta fish". Acute thoracolumbar kyphosis is a common deformity that manifests as vertebral humping, often before the age of two. It affects around three vertebrae, with the apical vertebra having anterior wedging and being posteriorly displaced.^19,30,31^ Lumbar stenosis is rare but can occur due to discal retropulsion. Although scoliosis may be present, it is much rarer and, when present, it is always associated with kyphosis.^19,30^ (Figure 9)

### FINAL CONSIDERATIONS

MPS are a group of skeletal dysplasias characterized by disproportionate short stature, usually rhizomelic. This condition comprises 15 types divided into seven phenotypes according to the specific enzyme deficiency. MPS present several osteoarticular alterations known as multiple dysostosis (disostosis multiplex). The main alterations include cervical instability and stenosis, platyspondyly and acute thoracolumbar kyphosis, claw hands and feet, valgus knees, hip dysplasia, and long bone deformities. Many of these characteristics are not specific to MPS and are also present in other skeletal dysplasias, but knowledge of the details of osteoarticular radiology is essential to avoid delays and diagnostic errors that can allow the patient's evolution to irreversible and even lethal conditions.

This report presents the main orthopedic and radiographic aspects in the limbs and spine of patients with mucopolysaccharidoses, emphasizing the role of these alterations in early diagnosis. The scarcity of similar studies certainly makes it difficult to correctly identify suspected patients since MPS are rare diseases with multiple differential diagnoses in the field of skeletal dysplasias, often leading to errors of evaluation. This article represents an important contribution to improving the suspicion, screening, and identification of MPS by pediatricians, geneticists, and rheumatologists who are usually unfamiliar with the details of orthopedic radiology.
